# Igbo Speech Surrogacy: Preliminary Findings Based on the Oja Flute

**DOI:** 10.3389/fpsyg.2021.653068

**Published:** 2021-06-14

**Authors:** Aaron Carter-Ényì, Nnaemeka C. Amadi, Quintina Carter-Ényì, Charles Chukwudozie, Jude Nwankwo, Ebruphiyor Omodoro

**Affiliations:** ^1^Morehouse College, Atlanta, GA, United States; ^2^University of Nigeria Nsukka, Nsukka, Enugu, Nigeria; ^3^University of Georgia, Athens, GA, United States

**Keywords:** speech surrogacy, tone language processing, speech rhythms, music analysis, contour theory, flute acoustics, Nigeria, Igbo

## Abstract

This research report presents analyses of recordings from the Ìgbò culture of southeastern Nigeria of an *ọ̀jà* flute player, a female speaker, and a male speaker. After a prepared performance, the participants completed two tasks: (1) mapping speech to flute playing and (2) identifying phrases played on the flute. Contour analysis is applied to annotated recordings to study the mapping of speech tone and rhythm from voice to instrument in parallel utterances by the three participants (male, female, and flute). Response time between the flute playing and spoken phrase identification indicates each prompt’s relative clarity. Using a limited but not predetermined inventory of related praise epithets, participants successfully converted speech to music and music to speech. In the conversion of speech to music, we found that declination was not part of the mapping, indicating it is a phonetic artifact of speech and does not carry a functional load. In identifying surrogate phrases played on the flute (music to speech), we found that dialectical variation caused some misidentification because idioms known in one area of the Igbo dialect cluster are not necessarily known throughout the region. However, *òòjà* speech surrogacy is found throughout the region. Possibilities and predictions for further research are presented.

## Introduction

We present preliminary findings from a computer-assisted study of Ìgbò *ọ̀jà* speech surrogacy based on a 30-min participant-observation session recorded at the University of Nigeria Nsukka on November 2, 2020. In the session, an *ọ̀jà* flute player, a female speaker, and a male speaker gave prepared performances. Then, the performance participants were asked by the researchers to complete two tasks:

(1) Mapping of speech to flute playing: the male speaker spoke a phrase praising the woman, the woman repeated, and then the flute played it. The participants repeated the process 16 times. The number of repetitions was not specified when the task was described.
(2) The recognition of phrases played on the flute: the flute player played a common phrase for the male speaker to identify. After a primer, this task was performed 18 times.

The Ìgbò language is spoken in southeastern and southern Nigeria, primarily in the states of Abia, Anambra, Ebonyi, Enugu, and Imo. It belongs to the Benue-Congo branch of the Niger-Congo family. Ìgbò is a two-tone language with downstep. Because of the relatively small number of tone levels–two levels, while many in Nigeria have three or more levels–Maddieson (2013) classifies it as a simple tone system in the World Atlas of Language Structures. However, previous research on Ìgbò and Yorùbá suggests that the functional load of tone in Ìgbò may be higher than that of Yorùbá. A comparison of two widely available dictionaries [Williamson’s (1972) Ìgbò Dictionary and the University of Ibadan’s Yorùbá Dictionary] revealed that 60% of disyllable entries formed minimal pairs in Ìgbò. In contrast, only 48% of disyllable entries in Yorùbá formed minimal pairs (Carter-Enyi, 2016).

*Ọ̀jà* is a small wooden high-pitched flute, approximately seven inches (18 cm) in length, indigenous to the Ìgbò people (Nwachukwu, 1997). Its usage is vast, but Lo-Bamijoko (1987) notes that it is used “more for chanting than for singing.” Lo-Bamijoko (1987) defines chanting as an “extended form of speaking,” more commonly known as speech surrogacy. The *Ọ̀jà* is played for the Ígweē (traditional ruler), notable chiefs or influential people in the community for entertainment, praise-singing, or relaxation. It may also take on a more important role during life-cycle celebrations such as naming ceremonies or marrying a new wife. The *ọ̀jà* may be seen as the soul of Ìgbò cultural music. The instrument is used to sing laments for the dead. In Ìgbò myths, the *ọ̀jà* is believed to possess spiritual power capable of even raising the dead. It is played for the *mmoọnwu* (masquerade representing spirit manifestation) as a morale booster during public displays. In recent times, composers use the instrument for soundtracks of Nollywood movies. It is sometimes described as “the oil with which Ìgbò music is eaten.” The sound energizes the weak and calls up the very aged to jump up in strength as they dance to its calls. In summary, *ọ̀jà* is a musical instrument of immense cultural significance among the Ìgbò people. In recent years, Christian Onyeji (2006, 2016) of the University of Nigeria Nsukka has advocated for the *ọ̀jà* and other Igbo instruments as mediums for art music composition. Nwachukwu (1997) is a detailed acoustic and organological study of the instrument.

Like Lo-Bamijoko, we assume that the “chanting” (language-based) mode of the *ọ̀jà* is primary to the instrument’s performance practice. Our aim was to collect data on the chanting mode, which would serve for further explorations of this under-studied genre. We recorded a participant-observation session to examine the mapping of speech to flute and flute to speech. This brief research report summarizes the findings from the computer-assisted analyses of these recordings.

## Materials and Methods

CC is a retired lecturer in African Studies at the University of Nigeria Nsukka. He is known in the community by nicknames, including “Akionu” and “Member.” Smartphone videos of his dancing are popular on social media^1^. He hosts a weekly radio show on the university radio station (Lion FM) on Ìgbò culture entirely in the Ìgbò language.

Mr. Chukwudozie approached Aaron Carter-Ényì about recording an òjà performance for the Africana Digital Ethnography Project (ADEPt, radar.auctr.edu/adept). Three videos are available on YouTube from the recording session: (1) Igbu Ọ̀jà (Playing the Flute): “Igwe O, Igwe^2^;” (2) Ìgbò Woman praised by the Ọ̀jà flute^3^; and (3) Ọ̀jà Phrase Identification^4^. In the recorded performance, Bartholomew Ogbu (*ọ̀jà* player), Chinyelum Ewelum, and Mr. Chukwudozie demonstrated *ọ̀jà* praise-singing for an Ìgwē (king) and Lọ̀lọ̀ (queen), observed in videos 1 and 2, respectively. The prepared performance included the flute “speaking,” playing common praise phrases on the flute by replicating the pitch and rhythm contours of speech.

After the group completed their prepared performance, they were asked to perform specific tasks. Although the prepared performance demonstrated the *ọ̀jà’s* capacity for surrogacy, articulating words such as “Ígwē” (king), the researchers deemed it necessary to have a closer comparison of equivalent phrases. Because the performance participants were aware of the *ọ̀jà’s* capacity for surrogacy, they could respond to specific tasks involving the transfer of speech to music and music to speech. The two tasks were (1) mapping speech to flute playing and (2) identifying phrases played on the flute. We describe the performance processes in detail with analysis in the following sections.

**FIGURE 1:**
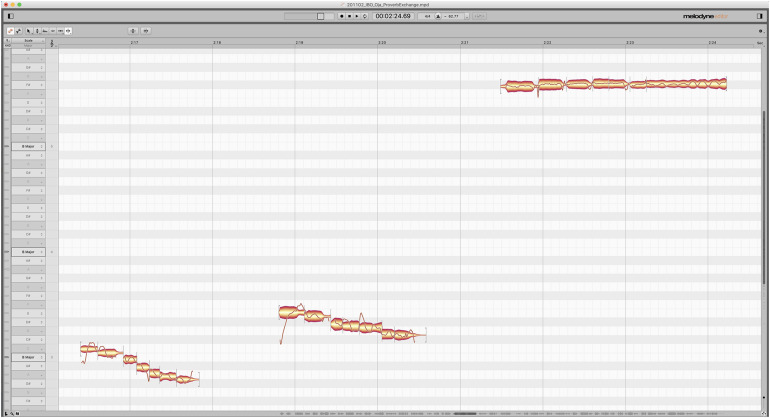
Task 1–Phrase 7 in Melodyne with male voice **(left)**, female voice **(middle)**, and *ọ̀jà* **(right)**.

For Videos 1 and 2, CC handwrote the transcriptions and translations of video excerpts. NA and Ugonna Okonkwo entered his written text as timed-text captions in YouTube Studio. Quintina Carter-Ényì completed all stages of language annotation for Video 3. The timed-text Igbo and English captions completed in YouTube Studio were then downloaded in the sub-rip title format (.srt) and imported into ELAN. However, this report focuses on the pitch and time domain, not segmental phones (phonemes). Aaron Carter-Ényì made additional annotations in Celemony’s Melodyne Editor (see Figure 1), which encodes pitch and timing information in MIDI format (where C4 = 60, C5 = 72, etc.). MIDI data (.mid) is interoperable with many software from MATLAB to Logic Pro. The Melodyne annotation was the final stage of annotation.

## Results: Task 1: Cross-Domain Mapping of Speech to Music

In discussion with the participants, we agreed that the “king” (Chukwudozie) would speak a praise epithet (e.g., “Ńné múrú-ọ̀rà” meaning “mother of a multitude”), the “queen” (Ewelum) would repeat it, and finally, the flutist (Ogbu) would play it. Everyone knew that the phrases would praise women, especially mothers. Every phrase belonged to a standard inventory of praise epithets (see Barber, 1991). The participants did not rehearse the phrases or their order. This task is available on YouTube as “Ìgbò Woman praised by the ọ̀jà flute (see footnote text 3).”

The video starts with an *ọ̀jà* introduction (timecode 00:00) and is followed by vocalizations by Chukwudozie portraying the Ìgwē (timecode 01:02). The praise sayings begin at timecode 01:20. All 16 of the phrases are in celebration of motherhood. Women in Ìgbòland are celebrated and praised because they are seen as the pillars of the home. The woman’s ability to manage the household and her husband’s wealth is all captured in the praises. The phrases highlight the woman’s qualities and characteristics, including the woman’s ability to bear, breastfeed, and raise a child. They also refer to the physical attributes of women, such as beauty and shapeliness.

We analyzed data from the first task to evaluate the similarity of the pitch and rhythm content between the three versions (male, female, and flute) for each of the 16 phrases. Specifically, an implementation of musical contour theory was applied to computer-assisted melodic transcriptions produced using Melodyne software. A script written in MATLAB calculated Quinn’s (1997) contour similarity to assess the similarity of the male speaker’s speech and the *ọ̀jà* interpretation (script attached, “Frontiers.m”). This analysis addresses the mapping of speech tone and rhythm to *ọ̀jà* playing through comparing combinatorial matrices of pairwise pitch height comparisons (or segment durations in seconds in the case of rhythm).

**FIGURE 2:**
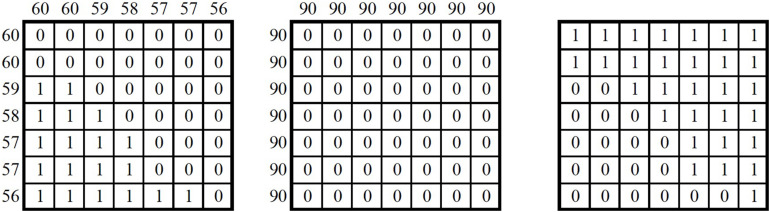
Contour matrices for the pitch of Task 1–Phrase 7 with male speech demonstration **(left)**, *ọ̀jà* interpretation **(middle)**, and similarity matrix **(right)**.

In Figure 2, at the left, each pitch height (60 = C4/Middle C) is compared to every other pitch height in the melodic segment. In this case, there are seven “notes.” The “melody” of the male voice speaking the phrase starts at 60 (C4) and gradually descends to 56 (Ab3). Quinn’s method (1997) codes binary pairwise comparisons as “1” for greater than and “0” for less than or equal. We compared the notes at the top of the columns to the notes along the left side. This yields self-comparisons (0 for equal) along the central diagonal from the top left to bottom right. The middle matrix is for the *ọ̀jà* interpretation. All of the notes are at the same pitch height (90 or F#6). All of the pairwise comparisons are “0” because all of the notes are equal. The rightmost matrix measures similarity between the speech matrix and music matrix. In this case, “1” indicates a match, and “0” indicates a non-match for each respective cell. Out of the 16 phrases recorded, annotated, and analyzed, Phrase 7 (see Figures 1, 2) had the lowest pitch contour similarity between speech and music, 61.2%. Notably, the phrase is on a single tone “Ónyé áká ghárá-ghárá” (all high tone level). This result suggests that declination is not necessarily part of the mapping from speech to music. The contrast in phrase declination between speech and music can be observed aurally by listening closely to Phrase 7 in the audio of the YouTube video linked above (timecode 02:16) and visually by examining the Melodyne transcription in Figure 1.

Although flute playing is studied here, not singing, this exclusion of declination effects is not limited to surrogacy. Chanted or sung realization of tones may also avoid declination (Carter-Enyi, 2016). The single-tone phrase; none of the other phrases consisted of a single tone. 11 out of 16 of the phrases had high pitch contour similarity, with above 80% of pairwise comparisons matching (see the second to last column, “Pitch,” in Table 1). The difference between the phrase declination characteristic of speech and the stable pitch height characteristic of music is not so pronounced in a phrase where the speech tone is more varied and the contour more complex.

**TABLE 1:** Results for Task 1 (speech to music).

#	Time	Ìgbò transcription	English gloss	Segments	Pitch	Rhythm
1	1:19	Nìné múrú-ọ̀rà	Mother of a multitude	6	0.917	0.611
2	1:27	Álá nàzù ńwā	Breast that feeds the babies	6	0.917	0.389
3	1:35	Óchìé díkē-ńnēm	My strong mother	5	0.880	1.000
4	1:44	Éléléb’úkwù è gbù’éwū	The waist that deserves to be celebrated with the killing of a goat	7	0.918	0.959
5	1:54	Ọ̀zùlù éké, zùọ̀ Óriè, zùọ̀ Áfọ̀, zúọ̀ Ǹkwò	She sells on Eke, Orie, Afor, and Nkwo market days	10	0.740	0.800
6	2:07	Ńné ńnọ̀ọ̀má	Mother, good mother	5	0.880	0.840
7	2:16	Ónyé áká ghárá-ghárá	A resourceful person	7	0.612	0.796
8	2:25	Ọ̀mụ̀rụ̀ Órìe, mụ́ọ́ Àfọ̀, mụ́ọ́ Ǹkwọ́	She gave birth to Orie, Afor and Nkwo	8	0.766	0.563
9	2:37	Ọ̀dị̀ dí yé ḿmā	Her husband’s delight	6	0.639	0.278
10	2:44	Ódózī àkụ̀ dí yá	The manager of her husband’s wealth	7	0.898	0.592
11	2:53	Ùgòó dí yá	The glory of her husband	5	0.760	0.520
12	3:01	Ézē nwáānyì	Queen mother	4	0.875	0.750
13	3:08	Ọ̀chílụ̀ ọ́zụ́ọ́	Trainer of all	6	0.889	0.611
14	3:16	Ágbàrà k’iìbeè yà	A woman greater than other women	6	0.944	0.778
15	3:24	Ńné ọ̀máááá!	A virtuous woman	3	1.000	1.000
16	3:33	Iì gàdị́ ńdụ́ rúé m̀gbè ébiÌghèbì	You will live forever and ever	10	0.880	0.540

**FIGURE 3:**
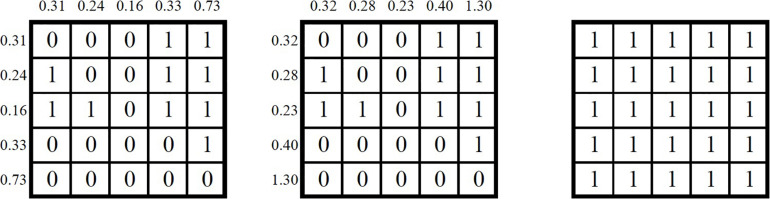
Contour matrices for the rhythm of Task 1–Phrase 3 with male speech demonstration **(left)**, *ọ̀jà* interpretation **(middle)**, and similarity matrix **(right)**.

We also applied contour similarity metrics to rhythm, specifically duration in seconds (the last column, “Rhythm,” of Table 1). Figure 3 shows the application to segment durations in hundredths of a second. Similarly, the binaries are coded as “1” when the column duration is greater than the row duration, or “0” if it is equal to or less than for the first two matrices (speech on the left and *ọ̀jà* in the middle). On the right, we compare the first two matrices, yielding all “1” values because all entries in the first two matrices match each other, yielding 100% similarity.

## Results: Task 2: Phrase-Level Identification of Ọ̀jà Surrogacy

Task 2 is available on YouTube as “Ọ̀jà Phrase Identification (see footnote text 4).” The second task assesses the intelligibility of speech surrogacy on *òòjaÌ*. Response time between the end of the flute phrase and the phrase’s identification by the speaker (Chukwudozie) indicates the ease with which the speaker can identify each specific phrase. The response time is measured as the time between the end of the *ọ̀jà* phrase and the start of the speaker’s identification. In general, the speaker quickly identified the phrase demonstrated by the *ọ̀jà*. Chukwudozie correctly identified 14 out of 18 phrases with a mean response time of 0.5 s. The *ọ̀jà* player had to prime the topic twice (the first two phrases) before the Ígwē (Chukwudozie) could start identifying. Only three phrases were misidentified. Of particular note is “Óbòdò dìkē o!” which means “Strong city,” when the *ọ̀jà* player intended “Peace be with you” consistent with utterances correctly identified later in the task. Also, dialect seems to be a factor because “Déèjé nù o!” (which is a common phrase in Enugu state) required some prompting from the *ọ̀jà* player (from Enugu state) for the Iìgwē (from Anambra state) to recognize it.

## Discussion

While the Yorùbá *dùndún* (talking drum) is the most iconic speech surrogate in Nigeria, perhaps West Africa, speech surrogates are found in many other Niger-Congo cultures, notably the Ìgbò. Awareness of speech surrogacy is embedded in Ìgbò culture and practiced on several indigenous instruments, notably the *ọ̀jà* (small wooden flute) and *ùfìè* (large log drum). Our preliminary study of the *ọ̀jà* suggests that the mapping from speech to music is more easily accomplished than the mapping from music to speech. While we cannot generalize this finding based on one small group of participants, this outcome is logical because there is a loss of information in the mapping from speech to music, namely the segmental phonemes. The recognition of the musical phrases as speech requires the reconstruction of missing information from an inventory of known idioms. It is likely that the speech phrases come from a limited inventory and may need to be associated with musical mapping through experience. They may not be recognizable to fluent speakers without significant cultural experience with surrogacy and ọ̀jà surrogacy specifically.

We found that there is considerable precision in the representation of both pitch and rhythm in Ìgbò *ọ̀jà* surrogacy, similar to Seifart et al.’s (2018) study of Amazonian Bora drumming. Tonal stability across Igbo dialects was first proposed by Emananjo (1978). According to Clark (1990), variation in segmental phonemes (such as/r/and/l/) is common between dialects, but tonemes are usually consistent in analogous phrases. Toneme consistency made it possible for the *ọ̀jà* artist (from Enugu) to communicate on his instrument with a man from Anambra state. However, when the flute spoke a common phrase in the Enugu dialect, Chukwudozie (the respondent) did not readily identify it. Observation and analysis of Task 2 suggest some characteristics of Ìgbò *ọ̀jà* speech surrogacy. Toneme consistency across dialects makes it possible for *ọ̀jà* speech surrogacy (which represents the pitch and rhythm of speech) to be communicative across dialects. Because it is idioms that are usually “spoken” by instruments, unless the idiom is known across dialects, the pitch and rhythm pattern will not be familiar.

Regarding Task 1, we observe that speech declination is not part of the mapping from speech to music. This result is consistent with observations of singing (Carter-Enyi, 2016) and suggests that declination is purely an effect of production, which does not seem to affect intelligibility. This cumulative evidence supports the position that declination is not a phonological aspect of language even in a “terraced” tone language such as Ìgbò.

Based on 6 min of recordings, these preliminary findings provide a basis for future research predictions. However, much more work must be done to determine the extent to which these observations may be generalized to other instruments and even different cultures. If we conducted a more extensive study with multiple participants responding to Task 2 (identification of surrogate phrases), we predict that participants will most readily identify stereotyped phrases. Likely, single words out of context cannot be identified. Even phrases without a larger context (e.g., a topic such as praise of a woman or king) are hard to identify.

## Data Availability Statement

The datasets presented in this study can be found in the Africana Digital Ethnography Project (ADEPt) collection of the Repository of AUC Digital collections, Archives and Research: https://radar.auctr.edu/adept.

## Ethics Statement

The studies involving human participants were reviewed and approved by University of Nigeria Nsukka, International Directorate. The participants provided their written informed consent to participate in the audiovisual recordings collected for this study. Written informed consent was obtained from the individuals for the publication of the audiovisual recordings referenced by this article.

## Author Contributions

ACÉ conducted the participant research, recorded and edited the videos, did the musical transcription, completed the data analysis, and drafted the article. QCÉ completed transcription and translation of one of the two videos analyzed, and also interpreted the data qualitatively as well as discussing extensively with the lead author. JN conducted ongoing speech surrogate research with the lead author and contributed text on both Igbo culture and the Oja flute in Igbo culture. NA prepared captions and the description for one of the videos which became part of the basis for a brief section of the report. EO assisted the lead author in recording the session and communicating with the participants. All authors contributed to the article and approved the submitted version.

## Conflict of Interest

The authors declare that the research was conducted in the absence of any commercial or financial relationships that could be construed as a potential conflict of interest.

## Footnotes

1 https://twitter.com/Gidi_Traffic/status/1237310343266938880

2 http://hdl.handle.net/20.500.12322/adept.ibo:0013

3 http://hdl.handle.net/20.500.12322/adept.ibo:0014

4 http://hdl.handle.net/20.500.12322/adept.ibo:0015
