# Supplementary material for: Igbo Speech Surrogacy: Preliminary Findings Based on the Oja Flute
Source: Front Psychol. 2021 Jun 14;12:653068. doi: 10.3389/fpsyg.2021.653068 (PMC8238117; doi:10.3389/fpsyg.2021.653068)
Supplement: Supplementary file 1 [file Table_1.DOCX]

Music-to-Speech Results

| Ọ̀jà Start | Ọ̀jà End | Duration | Speaker Start | Response Time | Identified Ìgbò | English Gloss | Notes |
| --- | --- | --- | --- | --- | --- | --- | --- |
| 00:03.2 | 00:07.4 | 00:04.2 | 00:08.0 | 00:00.6 | Ónyọ́chá, é kèlém gí! Bíá k’ànyí jégídé | White person, I greet you | Prompted |
| 00:12.4 | 00:14.1 | 00:01.7 | 00:15.8 | 00:01.7 | Bìá k’ànyí gùrígídé égwū | Come, let us play together | Prompted |
| 00:17.3 | 00:20.4 | 00:03.1 | 00:20.6 | 00:00.2 | Ónyọ́chá, k’ànyí gùríbé égwū, K’ànyí gùrígídébé égwū | White person, let’s play together; let’s play together |  |
| 00:22.7 | 00:23.9 | 00:01.2 | 00:24.2 | 00:00.3 | K'ànyí gùríbé égwū, K'ànyí gùríbé égwū | Let's play together; let's play together |  |
| 00:26.7 | 00:27.8 | 00:01.2 | 00:28.2 | 00:00.3 | Màkà ùdó g’ààdí | Hence, peace will reign |  |
| 00:29.7 | 00:31.1 | 00:01.3 | 00:31.2 | 00:00.1 | K’ùdó wèé dí | So that peace would reign |  |
| 00:32.5 | 00:33.7 | 00:01.2 | 00:33.8 | 00:00.1 | Mà k’údó díl’ányī | So that we would be at peace |  |
| 00:35.8 | 00:38.0 | 00:02.1 | 00:38.6 | 00:00.6 | Ónyọ́chá, déèjé nù o! | Whiteman, welcome! | Prompted |
| 00:41.1 | 00:43.9 | 00:02.8 | 00:44.9 | 00:01.0 | Òbòdò díkē o! | Strong city | Incorrect; sounds like “peace be with you” |
| 00:46.9 | 00:50.6 | 00:03.6 | N/A | N/A | N/A |  | No response |
| 00:52.0 | 00:55.5 | 00:03.6 | 00:56.3 | 00:00.8 | Í bìàlù nà báānyí k’ùdó wèé dí… | You have come to our land so that there will be peace… |  |
| 00:56.3 | 00:58.3 | 00:02.0 | 00:59.1 | 00:00.8 | kà ògànírú wèé búrú nk’ányī | so that progress will be ours |  |
| 01:03.5 | 01:05.7 | 00:02.2 | 01:05.8 | 00:00.1 | N'ọyà à dílí gí ḿmā! | And it will be well with you! |  |
| 01:07.7 | 01:09.9 | 00:02.2 | 01:10.1 | 00:00.2 | Ọ́yà à díl'ányí ḿmā! | It will be well with you! | * |
| 01:11.7 | 01:13.7 | 00:02.0 | 01:13.9 | 00:00.2 | Kà ànyí wèlíté óbì énū | Let us raise our hearts | same tune as * with a different interpretation |
| 01:14.8 | 01:16.4 | 00:01.6 | 01:16.6 | 00:00.2 | K’ùdó wèé díl’ányī | so that peace would reign | ** |
| 01:17.4 | 01:18.4 | 00:01.1 | 01:18.4 | 00:00.0 | Kà ànyí-ńchá wèé wúlú òfú | So we all can become one |  |
| 01:18.9 | 01:20.1 | 00:01.2 | 01:20.4 | 00:00.2 | K’ùdó díl’ányī | so that we will be at peace | same tune as ** with a different interpretation |
| 01:20.4 | 01:21.7 | 00:01.4 | 01:22.0 | 00:00.2 | Kà òbòdò níīlé nwé ògànírū | Let all nations move forward (progress) |  |
| 01:23.9 | 01:26.8 | 00:03.0 | 01:27.2 | 00:00.4 | Ónyé métú nwáńnē yé, nwáńnē yé è métú íbè yé | One touches the brother, and that one touches another |  |
| 01:27.2 | 01:30.0 | 00:02.8 | 01:31.0 | 00:01.0 | K’ùdó wèé dí n’òbòdò | So that peace would reign |  |
|  | Mean: | 00:02.2 | Mean: | 00:00.5 |  |  |  |
